# Temperature moderates impact of formulated moxidectin on seed germination of three temperate grassland species

**DOI:** 10.1371/journal.pone.0277865

**Published:** 2022-11-21

**Authors:** Carsten Eichberg, Alwin M. Hartman, Alexandra-Marie Kronenberger, Rolf-Alexander Düring, Tobias W. Donath

**Affiliations:** 1 Geobotany, Regional and Environmental Sciences, University of Trier, Trier, Germany; 2 Analytical and Ecological Chemistry, Regional and Environmental Sciences, University of Trier, Trier, Germany; 3 Institute of Soil Science and Soil Conservation, Justus Liebig University, Gießen, Germany; 4 Department of Landscape Ecology, Institute for Natural Resource Conservation, Kiel University, Kiel, Germany; Savitribai Phule Pune University, INDIA

## Abstract

Formulations of macrocyclic lactone anthelmintics such as moxidectin are regularly administered to sheep to combat parasites. A disadvantage of these pharmaceuticals are their side effects on non-target organisms when entering the environment. Little is known about anthelmintic effects on plant reproduction and whether the effects depend on environmental factors. For ecological and methodological reasons, we aimed at testing whether temperature affects the efficacy of a common moxidectin-based formulation on seed germination. We carried out a germination experiment including three typical species of temperate European grasslands (*Centaurea jacea*, *Galium mollugo*, *Plantago lanceolata*). We applied three temperature regimes (15/5, 20/10, 30/20°C), and a four-level dilution series (1:100–1:800) of formulated moxidectin (i.e., Cydectin oral drench). These solutions represent seed-anthelmintic contacts in the digestive tract of sheep shortly after deworming. In addition, a control was carried out with purified water only. We regularly counted emerging seedlings and calculated final germination percentage, mean germination time and synchrony of germination. Formulated moxidectin significantly reduced percentage, speed and synchrony of germination. A 1:100 dilution of the formulation reduced germination percentage by a quarter and increased mean germination time by six days compared to the control. Temperature moderated effects of the anthelmintic drug on germination in all response variables and all species, but in different patterns and magnitudes (significant anthelmintic x temperature x species interactions). In all response variables, the two more extreme temperature regimes (15/5, 30/20°C) led to the strongest effects of formulated moxidectin. With respect to germination percentage, *G*. *mollugo* was more sensitive to formulated moxidectin at the warmest temperature regime, whereas *P*. *lanceolata* showed the highest sensitivity at the coldest regime. This study shows that it is important to consider temperature dependencies of the effects of pharmaceuticals on seed germination when conducting standardised germination experiments.

## Introduction

Seed germination is a vulnerable stage in the plant life cycle [1]. The quantity and timing of germination in the field can impact plant population development and species composition of a plant community [2]. Many factors influence seed germination and in the natural habitat these factors act in combination [3,4]. Here, we focus on two factors that are of particular importance for germination of grassland species in cultural landscapes: air temperature and an anthelmintic drug as a representative of agricultural chemicals.

The massive use of agricultural chemicals in the European landscape is identified as one of the main drivers of biodiversity loss, including plant diversity [5–7]. Among agricultural contaminants, veterinary phamaceuticals play an important role encompassing several hundred active ingredients in the European Union [8]. An important group of veterinary phamaceuticals are anthelmintics which are used to control gastrointestinal parasites [9]. Endoparasites are a major cause of ill health and lower productivity in livestock [10], and therefore anthelmintics are regularly and worldwide administered to domestic livestock species. Their application is important to guarantee livestock welfare.

Among anthelmintics, macrocyclic lactones (MLs) play a pivotal role in the control of parasites in ruminant husbandry around the world [11–13]. MLs are either natural or chemically modified compounds produced by various soil bacteria of the genus *Streptomyces* [14]. They are relatively large and highly lipophilic substances, which are distributed expansively throughout the animal body and accumulate in fat tissues from where they are successively released [14,15]. MLs oscillate between tissues and the lumen of the gastro-intestinal tract and show long half-lives [11]. Due to their lipophilic character, MLs are almost completely associated with rumen particulate matter [14] and are excreted mainly via faeces [16]. A disadvantage of these drugs is their toxicity to non-target organisms. Many studies reported lethal or sublethal effects of MLs on decomposers, especially dung-inhabiting arthropods, with the consequences of reduced nutrient cycling and, ultimately, pasture quality [reviewed in 12,13]. Contrastingly, studies on phytotoxicity of MLs and their formulations are widely missing [9]. Recently, it has been shown that MLs can have effects on seed germination and root growth in seedlings [17,18]. Under natural conditions, these effects might have consequences for the population development of grassland plants and need further research.

Seeds of wild plant species come into contact with MLs in the digestive tract of livestock animals if they have been eaten and the animals have been dewormed. If deworming is done with a formulation and shortly after the seeds have been eaten, the seeds are not only exposed to the pure ML but also its adjuvants. These may also affect seed germination [19–21]. Eaten seeds stay on average about two days in the digestive tract of sheep, before they are excreted [22]. This time span is long enough to allow seeds absorption of water [23] and, consequently, of chemical substances dissolved in it. MLs show a high metabolic stability and and are mostly excreted in a non-metabolised form [13,24]. This means that at least the active ingredient of ML formulations potentially remains in contact with the eaten seeds in dung heaps.

The consumption and internal transport of seeds by livestock species (endozoochory) leads to the spatial distribution of seeds and–to some extent–the colonisation of new habitats or habitat sections. In grazed grassland, the endozoochorous transport of seeds by ungulates plays an important role for plant reproduction: Ungulates disperse high quantities of seeds [25], seeds from a large share of the regional species pool [26] and can help seeds in the establishment of the offspring by providing suitable microsites for colonisation [27]. This coupled ecological function of seed transport and seed-bed preparation by ungulates gets even more important in fragmented landscapes, where it is difficult for plant species of isolated populations to exchange seeds and establish the genetic information contained therein. Seed-dispersal limitation is a major constrain to grassland phytodiversity in the fragmented European landscape [28]. Mobile sheep flocks are one of the few seed vectors that potentially mitigate such dispersal limitations [29]. Sheep grazing has proven to be an important management tool for various grassland types in the European landscape [25] and is positively correlated with high species richness [30].

Among the environmental factors that control seed germination, temperature is of major importance [3,31]. Temperature can directly act on seed germination of non-dormant seeds by influencing the speed of biochemical reactions or indirectly by regulating seed dormancy [31–33]. A vast body of literature exists with respect to influences of temperature on seed germination. However, the influence of temperature on the effects of agricultural chemicals has rarely been studied, especially with respect to plant species. The few studies published mainly focus on aquatic plants [e.g. 34].

Temperature is an important signal, informing seeds about environmental conditions, whether they are suitable for seedling establishment [3]. Seeds show a high species-specifity in their temperature requirements; even species within the same habitat type can differ in their germination strategies [35]. On the other hand, grassland species and species with a wide distribution range, as studied here, show a wide range of suitable temperatures for germination [36,37]. As a mean temperature value for herabecous, non-weed species of open temperate habitat types that induces high germination percentages 19°C were calculated [23]. If the temperature is outside the optimal range of a species, it can cause additional stress to a plant individual which is exposed to a toxic environmental contaminant.

In this study, we expect seed germination to be affected by formulated moxidectin (Cydectin® 0.1% oral drench for the application at sheep) and that the size or sign of this effect depends on temperature. If there were to be effects, this would have both ecological and methodological implications. Since the reproduction success of a plant species depends not only on the total number of emerged seedlings of a seed cohort, but also on the timing of germination, we also analysed mean germination time (speed) and synchrony of germination (simultaneity) [38,39]. To test the effects of formulated moxidectin and temperature on seed germination, we carried out a germination experiment including three co-occuring species that are widespread across Central Europe and typical for mesophytic grasslands: *Centaurea jacea*, *Galium mollugo* and *Plantago lanceolata*.

We tested the following hypotheses:

1. Moxidectin oral formulation reduces the final percentage and synchrony of seed germination and it increases mean germination time, with higher concentrations leading to stronger effects.
2. Temperature alters the effects of moxidectin oral formulation in all response variables, with higher temperatures (30/20°C) enhancing the effects.

## Materials and methods

### Chemistry and pharmacology of test formulation

We tested the anthelmintic drug Cydectin® 0.1% oral drench for the application at sheep (Zoetis Deutschland GmbH, Berlin, Germany). Oral administration is the typical application route in small ruminants with long hair [40]. In this pharmaceutical, the active ingredient moxidectin (molecular weight: 640 g mol^-1^, log K_OW_: 4.8 [41]) is formualted together with eight adjuvants (package leaflet). In higher amounts these are: benzyl alcohol (a solving and conservative substance), butylated hydroxytoluene (an antioxidant) and disodium edetic acid (a stabilsing and chelating antioxidant). In agricultural practice, moxidectin is applied to sheep in a dose of 0.2 mg kg^-1^ body weight, independent of application route.

Moxidectin, a milbemycin ML, is a broad-spectrum endectocide used against various parasitic invertebrates from the phyla of nematodes and arthropods [11]. In invertebrates, MLs activate glutamate-gated chloride channels leading to an irreversible influx of chloride ions followed by membrane hyperpolarisation and muscle paralysis [42].

In our experiment, we studied Cydectin solutions with a dilution series corresponding to moxidectin concentrations in the range of 1.25–10 mg l^-1^. These values cover the upper range of concentrations of MLs reported for substrates (chyme, faeces) that are relevant for a seed endozoochorously dispersed by sheep. Since values for the concentrations of the adjuvants of Cydectin in environmental matrices are not available, we will refer exclusively to values reported for moxidectin itself and avermectin MLs. Avermectins have a similar structure and biological activity as milbemycins [13]. If a sheep is treated with Cydectin oral drench, a seed that is already in the rumen is exposed to a Cydectin solution with a moxidectin concentration of 1 g l^-1^. Much lower levels of MLs are reported for abomasal contents, ranging from 0.45 μg g^-1^ (0.5 d post treatment) to 0.03 μg g^-1^ (2 d p.t.) [15]. Generally, faeces showed much higher moxidectin concentrations than chyme, but reported values cover a broad range between 0.02 μg g^-1^ (40 h p.t. [43]) and 3.39 ppm (1 d p.t. [44]). To our knowledge, the highest reported value for the concentration of a ML in sheep faeces is 13.62 μg g^-1^ (ivermectin, 1 d p.t. [45]).

### Test species and seed material

We tested the seeds of three perennial herbaceous species: *Centaurea jacea* L. (Asteraceae), *Galium mollugo* L. (Rubiaceae) and *Plantago lanceolata* L. (Plantaginaceae). These species are typical representatives of temperate grasslands in Central Europe. The seeds of all tested species are naturally spread by sheep via passage through the gastrointestinal tract in viable condition [46,47]. In Europe, seed germination of the test species predominantly occurs in spring (*C*. *jacea*) or spring and autumn (*G*. *mollugo*, *P*. *lanceolata*) [48–50]. Seeds were obtained from a commercial supplier (Appels Wilde Samen GmbH, Darmstadt, Germany).

### Experimental design

A factorial experiment was established to study the effects of an anthelmintic drug (factor levels [k] = 5; purified water and 4 dilution levels of Cydectin), air temperature (k = 3) and species (k = 3) on seed germination. Five replicates were established per treatment combination, resulting in 225 experimental units in total. By adding specific amounts of purified water, four dilutions of Cydectin 0.1% oral drench were obtained (1:800, 1:400, 1:200, 1:100) corresponding to the following moxidectin concentrations: 1.25, 2.5, 5 and 10 mg l^-1^. In addition, a control was prepared with purified water only. We exposed the seeds to alternating 12 h light (200 μmol m^−2^ s^−1^ PAR) and 12 h dark periods in different climate chambers that generated three different temperature regimes (15/5, 20/10, 30/20°C).

In each species, 50 seeds were spread on one piece of filter paper in a glass Petri dish (9 cm Ø) and 5 ml of the respective treatment solution was applied. Glass material was used during all process stages of the production and use of the Cydectin solutions to minimise the adherence of the active ingredient to solid surfaces [23]. In order to reduce evaporation, Petri dishes were wrapped laterally with parafilm [23] and five Petri dishes were piled up in one transparent plastic bag.

Since most temperate perennial species profit from chilling for germination, including at least two of the test species (*G*. *mollugo*, *P*. *lanceolata* [23]), a cold wet stratification (5°C, darkness, 16-d period) was applied in a climate chamber to the used seeds prior to treatment. Initially, 2 ml of purified water was added to each dish. Twice a week a check was made in almost complete darkness to see whether the filter paper of each dish was still moist and, if not, small amounts of water were added to ensure that all seeds are completely soaked. No seed germinated during the stratification period. After stratification, the seeds of each Petri dish were carefully transferred to a new glass Petri dish with new filter paper before the treatment solution was poured over them.

Germinated seeds (appearance of radicle) were counted and removed regularly in a 3.5-d interval. Position of dishes within plastic bags and position of dish piles in the climate chamber was changed randomly at each counting date. Exposition time of the dishes in the climate chamber was five weeks, resulting in ten counting dates.

### Statistical analyses

We calculated final germination percentage (GP; %), mean germination time (MGT; days) and synchrony of germination (Z; dimensionless) for each replicate [38,39]. GP is the percentage of germinated seeds from the initial number of seeds, MGT is a measure of the weighted average length of time required for germination and Z indicates the germination variability during the experiment [38,39]. Z ranges from 0 to 1; the higher the values, the more synchronous the germination is.

To standardise the results across species (which varied in germination capacity), we have calculated logarithmic response ratios (LnRR [51]) for all dependent variables as LnRR = ln (x_trt_/${\bar{x}}_{c}$), where x_trt_ is the value of treated seeds and ${\bar{x}}_{c}$ is the mean value of the control. An effect of an independent variable was considered significant, when the 95% confidence interval did not overlap with zero [52]. LnRR cannot be calculated for replicates in which Z = 0, because the logarithm of zero is not defined. This was the case in 1 of 225 experimental units (*P*. *lanceolata*; 15/5°C; 1:200 dilution). This replicate was excluded from analysis when testing Z.

Analysis of variance (ANOVA) and Tukey HSD test were used to compare the LnRR among treatments. The predictor variables *species* (k = 3; *C*. *jacea*, *G*. *mollugo*, *P*. *lanceolata*), *anthelmintic* (k = 4; 1:100, 1:200, 1:400, 1:800 dilution) and *temperature* (k = 3; 15/5, 20/10, 30/20°C) were tested on the three response variables. In order to test the effects of the treatments at the species level, we carried out separate ANOVAs and Tukey HSD tests for each species. In each data set, normality and homogeneity of variances has been checked and confirmed visually by means of diagnostic plots. In order to check the data structures, interaction diagrams were also used (not shown). If the interaction diagrams gave strong evidence that there was an interaction of *anthelmintic* x *temperature*, then post-hoc tests were also carried out for marginally significant interactions (0.05 < p < 0.1; two cases: *C*. *jacea*: GP; *G*. *mollugo*: MGT). All statistical tests were conducted using SPSS 27.

## Results

### Germination percentage

**Fig 1:**
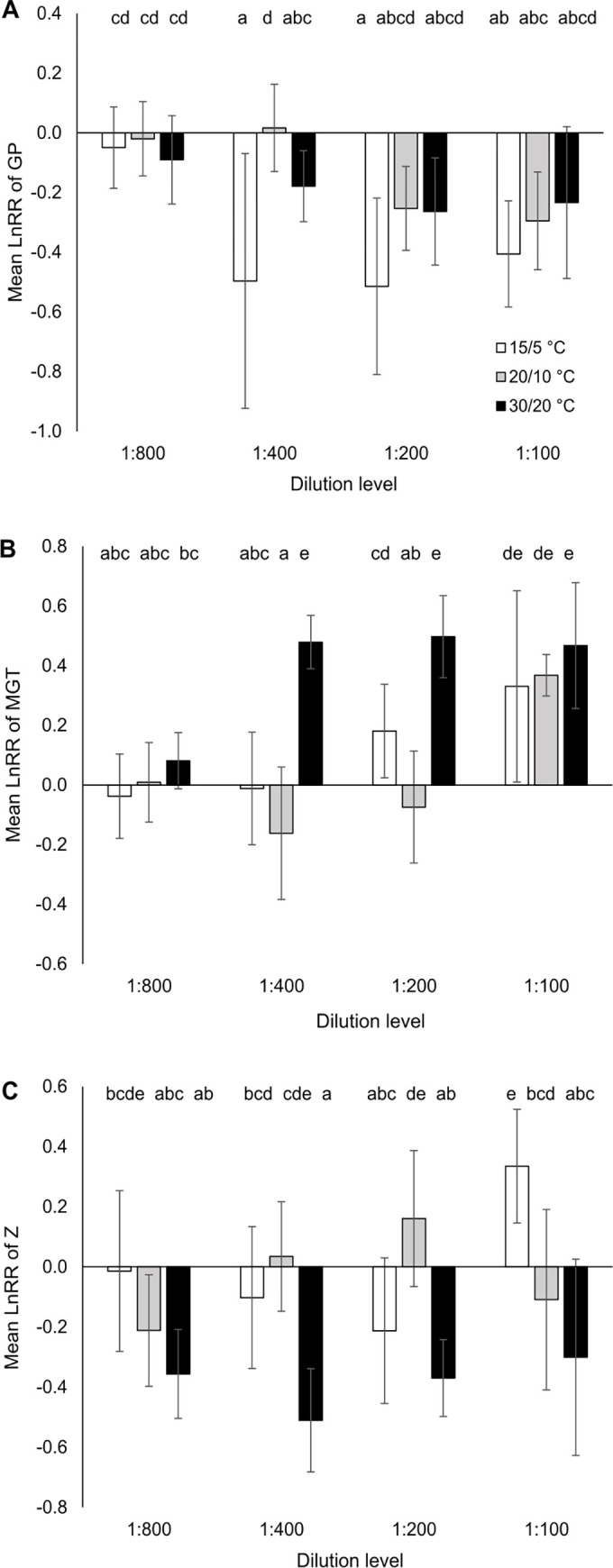
Mean (± 95% confidence intervals) natural-logarithm response ratio (LnRR) of A) germination percentage (GP), B) mean germination time (MGT) and C) synchrony of germination (Z) for a dilution series of the anthelmintic formulation Cydectin (moxidectin 0.1%) and three different temperature regimes (across species). Effect of formulation was considered significant when the 95% confidence interval did not overlap with zero. Different letters indicate significant differences between treatments (p < 0.05; Tukey HSD test).

**Fig 2:**
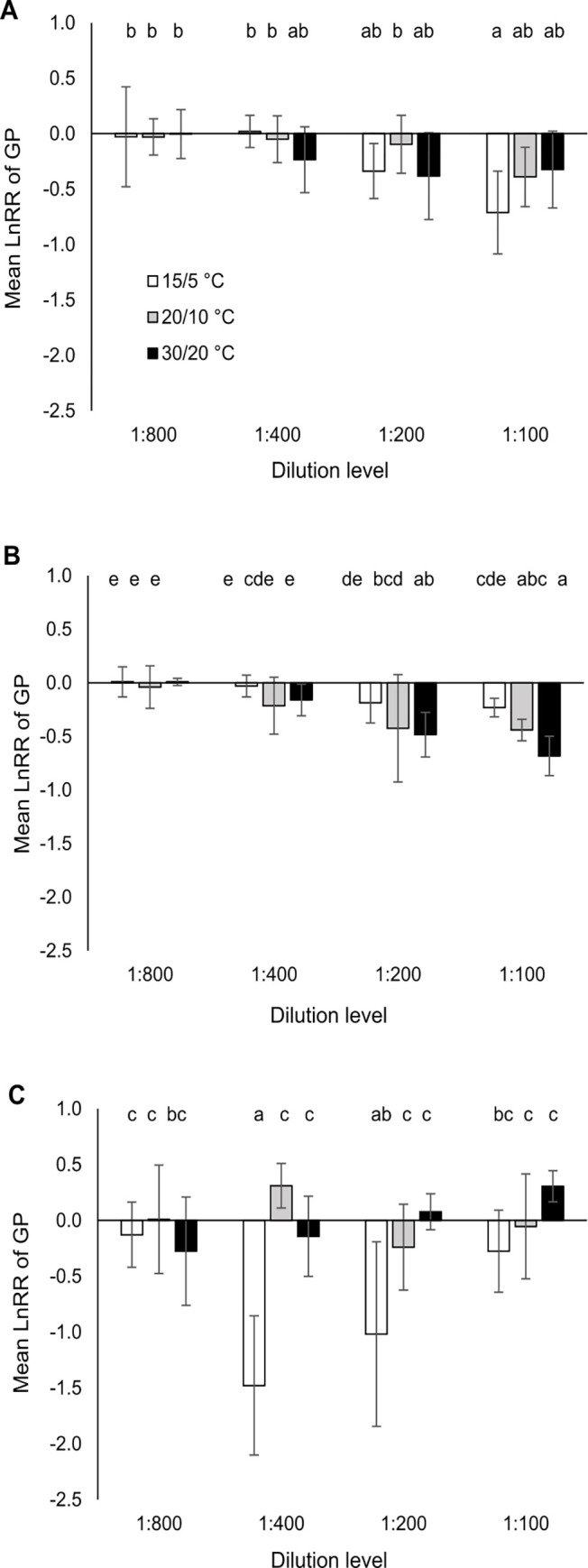
Mean (± 95% confidence intervals) natural-logarithm response ratio (LnRR) of germination percentage (GP) for a dilution series of the anthelmintic formulation Cydectin (moxidectin 0.1%) for three different temperature regimes and three test species (A: *Centaurea jacea*, B: *Galium mollugo*, C: *Plantago lanceolata*). Effect of formulation was considered significant when the 95% confidence interval did not overlap with zero. Different letters indicate significant differences between treatments (p < 0.05; Tukey HSD test).

For GP, all interaction terms were significant (Table 1), indicating the mutual influence of the three independent factors on one another. A three-way interaction of *species* x *anthelmintic* x *temperature* with high effect size (η^2^ = 0.38) was found. Thus, as expected, there was an influence of *temperature* on how *anthelmintic* affected GP, but this effect was species-dependent in terms of pattern and amplitude. Different responses of the tested species led to fluctuations in the across-species analysis and only a few significant differences between the factor level combinations (Fig 1A). Whereas *C*. *jacea* showed no significant differences between factor level combinations within a dilution level (Fig 2A), *G*. *mollugo* showed significant differences in the upper (Fig 2B) and *P*. *lanceolota* in the middle concentration range (Fig 2C). *G*. *mollugo* was more sensitive to formulated moxidetin at the warmest temperature regime than at the coldest regime, while *P*. *lanceolata* showed the highest sensitivity at the coldest regime. The reduction of GP in the middle concentration range was so strong in *P*. *lanceolata* compared to the control [75% (1:400); 58% (1:200)] that it shaped the response pattern of the across-species analysis (Figs 1A and 2C).

**Table 1:** Results of ANOVA on the effects of plant species, temperature regime and anthelmintic formulation (Cydectin, moxidectin 0.1%) on natural-logarithm response ratio (LnRR) of germination percentage (GP), mean germination time (MGT) and synchrony of germination (Z).

	LnRR of GP					LnRR of MGT					LnRR of Z				
	df	MS	F	p	η^2^	df	MS	F	p	η^2^	df	MS	F	p	η^2^
Intercept	1	9.679	148.352	<0.001	0.683	1	5.675	165.208	<0.001	0.534	1	3.513	39.442	<0.001	0.216
Species (S)	2	0.013	0.198	0.821	0.003	2	0.327	9.524	<0.001	0.117	2	0.737	8.270	<0.001	0.104
Temperature (T)	2	0.853	13.078	<0.001	0.154	2	1.961	57.097	<0.001	0.442	2	2.673	30.011	<0.001	0.296
Anthelmintic (A)	3	0.762	11.675	<0.001	0.196	3	1.148	33.419	<0.001	0.410	3	0.282	3.168	0.026	0.062
S x T	4	1.485	22.761	<0.001	0.387	4	1.432	41.681	<0.001	0.537	4	1.431	16.060	<0.001	0.310
S x A	6	0.564	8.652	<0.001	0.265	6	0.744	21.653	<0.001	0.474	6	0.828	9.290	<0.001	0.280
T x A	6	0.203	3.109	0.007	0.115	6	0.358	10.432	<0.001	0.303	6	0.558	6.259	<0.001	0.208
S x A x T	12	0.474	7.271	<0.001	0.377	12	0.094	2.728	0.002	0.185	12	0.197	2.213	0.014	0.157
Error	144	0.065				144	0.034				143	0.089			

df degrees of freedom, MS mean square, p error probability, η^2^ partial eta squared.

Across *anthelmintic*, *temperature* caused a maximum of GP in the 20/10°C treatment in all plant species. Across *temperature* and *species*, *anthelmintic* reduced GP, the effect decreasing with increasing dilution from 1:200 to 1:800 (Fig 1A). In the two weakest dilution levels (1:100, 1:200), a maximum reduction of GP by about a quarter was found compared to the control. In the upper concentration range (1:400–1:100), most *temperature* x *anthelmintic* combinations showed a significant reduction of GP compared to the control (no overlap of 95% confidence interval with zero line; Fig 1A).

### Timing of seed germination

For the timing variables (MGT, Z), all main effects and interaction terms were significant (Table 1). The interactions *species* x *temperature* (MGT: η^2^ = 0.54, Z: η^2^ = 0.31) and *temperature* x *anthelmintic* (MGT: η^2^ = 0.30; Z: η^2^ = 0.21) showed high effect sizes in both timing variables.

**Fig 3:**
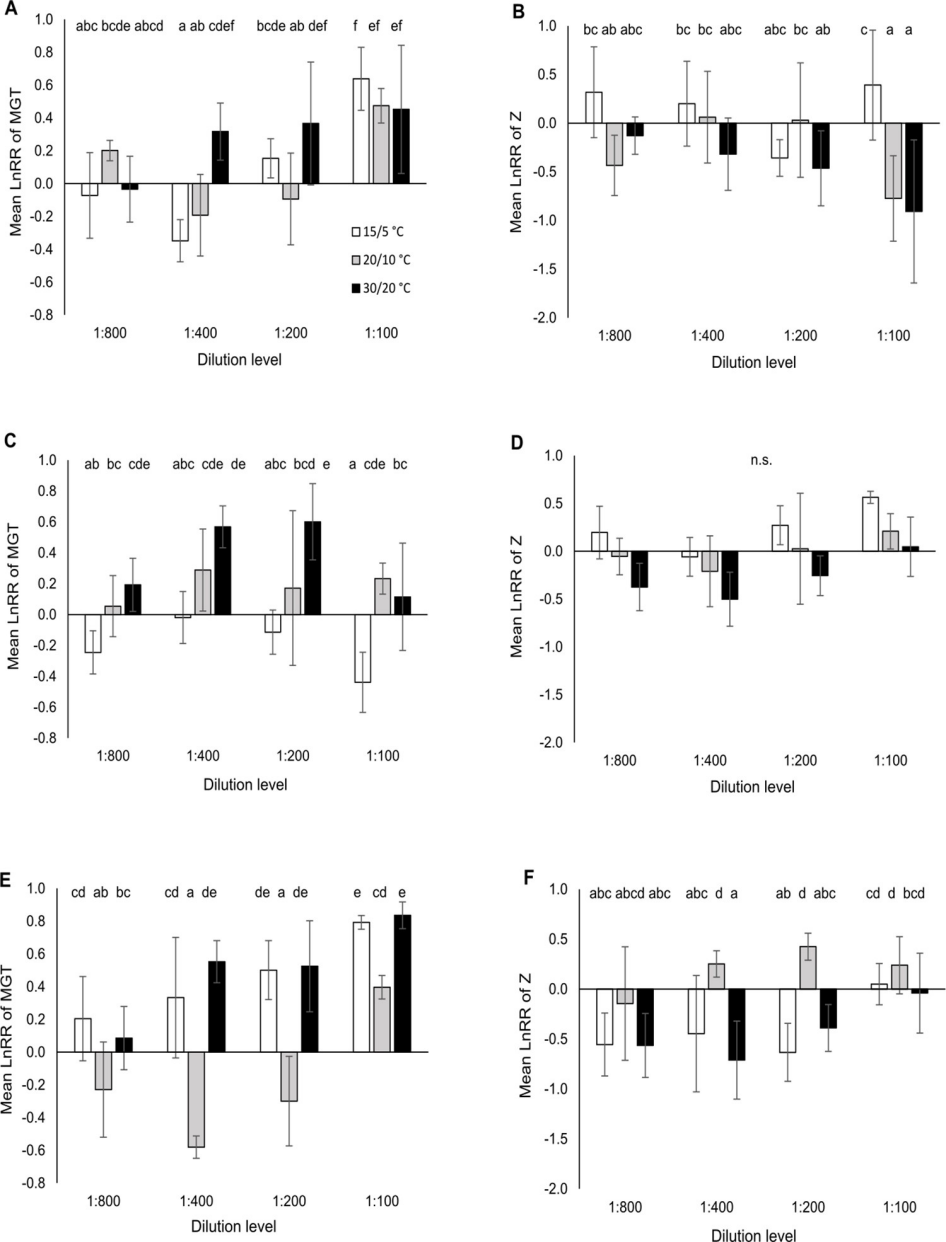
Mean (± 95% confidence intervals) natural-logarithm response ratio (LnRR) of the timing variables: Mean germination time (MGT) and synchrony of germination (Z) for a dilution series of the anthelmintic formulation Cydectin (moxidectin 0.1%) for three different temperature regimes and three test species (A,B: *Centaurea jacea*, C,D: *Galium mollugo*, E,F: *Plantago lanceolata*). Effect of formulation was considered significant when the 95% confidence interval did not overlap with zero. Different letters indicate significant differences between treatments (p < 0.05; Tukey HSD test).

Across *temperature* and *species*, MGT was increased by *anthelmintic*, the effect decreasing along the dilution series of 1:100 to 1:800 (Fig 1B). This pattern is mainly observed in *C*. *jacea* and *P*. *lanceolata* (Fig 3A and 3E). On average, a 1:100 dilution of formulated moxidectin increased MGT by six days compared to the control (increase by 67%). The warmest (*G*. *mollugo*, *P*. *lanceolata*; Fig 3C and 3E) or the coldest (*C*. *jacea*, Fig 3A) temperature regime led to the strongest effect of formulated moxidetin on MGT.

Across *species* and *temperature*, Z was lowered by *anthelmintic* with a maximal reduction at the 1:400 Cydectin dilution (18% lower than in the control) and lesser reductions in the 1:800 (13%) and 1:200 dilutions (7%). *C*. *jacea* deviated from this pattern showing the highest reduction at the highest concentration (Fig 3B). Similar to MGT, the two more extreme temperature regimes (15/5, 30/20°C) led to the strongest effects of formulated moxidectin (Figs 1C, 3B, 3D and 3F).

## Discussion

### Effects of formulated moxidectin on seed germination

We found that the sheep anthelmintic formulation Cydectin reduced final germination percentage, germination speed and synchrony of germination of temperate grassland species. These results corroborate earlier findings of germination experiments with moxidectin in formulated and non-formulated form [17]. In these previous experiments, we were able to show that the pure moxidectin also has a reducing effect on seed germination, albeit it was less effective than in formulation (Cydectin). An obvious explanation for this could be that the full effectiveness of moxidectin is only given if both the potentiating and the protective effect of its adjuvants is present. Without adjuvants moxidectin is prone to degradation by oxidation and UV light [41]. This might reduce moxidectin efficacy in germination experiments using the pure active ingredient and lead to an underestimation of its effect on eaten seeds. In addition, we found that also *in situ* lowering effects of formulated moxidectin on germination percentage can occur by means of a seed feeding experiment with sheep [17].

It has been shown that MLs, including moxidectin, can be absorbed by roots, distributed in the plant, partly metabolised and cause oxidative stress [53–55]. In a greenhouse pot experiment where soil was spiked with ivermectin, this could be found in the roots and leaves of soybean plants (*Glycine max*) but not in their fruits [55]. The cause of this inner barrier has not yet been clarified. It is also unclear whether moxidectin is able to pass the seed coat and diffuse into the embryo tissue when seeds are in the environment separately from the donor plant. By means of a fluorescent tracer [7-Amino-4-(trifluoromethyl)coumarin] its was shown that the ability of organic molecules to penetrate the seed coat of isolated seeds can depend on plant species [56]. In snap bean (*Phaseolus vulgaris*), the tracer was detected as well in the seed coat as in the embryo tissue indicating testa permeability, whereas in cucumber (*Cucumis sativus*) the tracer did not reach the embryo and was found only in the seed coat [56]. A semipermeable perisperm-endosperm envelope, present in cucumber seeds but not in bean seeds, could be causative for this difference [56]. The high lipophilicity and high molecular weights of MLs suggests that the passage through biological membranes is difficult for these molecules [56–58]. Future research must both clarify whether moxidectin and/or its adjuvants can reach the embro through closed seed coats and what are the receptors for molecules contained in anthelmintics in plants.

Here, we studied moxidectin in formulation, because this scenario is close to reality where eaten seeds in the digestive tract of sheep come into contact with a mixture of active ingredient and adjuvants. On the one hand, the adjuvants contained in a pharmaceutical formulation protect the active ingredient and increase its efficacy; on the other hand, adjuvants can cause toxic effects on organisms themselves. For instance, the two most abundandly represented adjuvants in the tested formulation, benzyl alcohol (BA) and butylated hydroxytoluene (BHT), can directly affect seed germination. BA has been shown to stimulate germination of dormant seeds of two millet species [19,20]. However, the BA concentrations used in these studies (10–300 mM) were 3- to 81-fold higher than the highest BA concentration studied here (3.7 mM BA in the 1:100 dilution of formulated moxidectin). An ether fraction of seeds of *Sapium sebiferum* that contained a high share of BHT (20%) reduced germination percentage of non-dormant cabbage seeds (*Brassica chinensis*) significantly [21]. However, the effect of BHT on germination percentage was only assumed because of its high proportion in the tested fraction of *S*. *sebiferum* seeds and because it inhibited root growth when applied as a pure BHT solution [21]. We assume that the adjuvants of anthelmintic formulations play a role in phytotoxicity of anthelmintic formulations on seed germination. However, there is a large gap of knowledge with respect to these substances and it is unclear whether and to what extent they may contribute to the found phytotoxic effects of anthelmintic formulations. Future experiments may also need to disentangle direct effects of adjuvants on seed germination from indirect effects (potentiation of effects of MLs).

The anthelmintic effects found in this study differed between the three tested plant species. Differences could be caused by specific seed-anatomical features, specific metabolisation pathways leading to differing metabolites with potential toxic effects and/or varying degrees of sensitivity to parent substances or metabolites. Species-specific responses to formulated moxidectin might lead to changing competitive relationships in grazed grasslands, accordingly, a shift in plant population sizes and, ultimately, changes in plant species composition. To ensure successful establishment of the offspring, plants depend on the successful dispersal of their seeds [59]. Not only for annual species, but also for perennial species, as studied here, it is important to successfully reproduce on the generative level in order to colonise new habitats and maintain genetic diversity. Sheep are effective seed vectors that enable plants to achieve this colonisation success [25]. However, due to economic restrictions, sheep farming in Europe has been on the decline for decades [60]. This is true especially for shepherded grazing, which is most associated with positive effects on the environment [61]. Therefore, deworming treatments might additionally limit the important seed dispersal function of roaming sheep flocks in the European cultural landscape. The species tested here are currently not threatened in Germany, but for two of them, *C*. *jacea* and *P*. *lanceolata*, significantly decreasing frequencies in German grasslands over the past 50 years were found [62].

Across species and temperature, two response variables showed a continuous decrease (GP) or increase (MGT) with concentration of formulated moxidectin, whereas Z was maximally reduced by formulated moxidectin in the middle concentration range. Therefore, hypothesis 1 could only partly be confirmed. All species were sensitive to formulated moxidectin but different species responses led to an inhomogenous response pattern. In GP and MGT, two species each showed roughly the expected relationship between concentration and effect, whereas in Z, only one species (*C*. *jacea*) tendencially showed the expected releationship. Non-linear dose response relationships are frequently found in studies dealing with effects of organic contaminants on plants [63,64]. The low-dose range often evokes other reactions of a plant individual to the stressor than the high-dose range and it is very important to include low-doses of a contaminant in ecotoxicological studies to achieve a realistic risk assessment [64].

A 1:100 dilution of formulated moxidectin increased MGT by six days compared to the control. In productive habitats, a delayed germination is disadvantageous for plant individuals, because with ongoing season light competition increases and establishment success decreases [2]. The plant species studied here occur mainly in medium-, and, to a lesser extent, in high-productive grasslands. In addition, a delayed germination, i.e. higher MGT, poses the risk that seedling development does not correspond to climate patterns species are adapted to [65]. Later germination in spring reduces biomass development above- and belowground compared to older seedlings [66]. These effects may be so strong that seedlings germinating later in spring can not develop sufficient root-soil contact to endure subsequent warmer and dryer summer periods and therefore may have a lower survival probability.

### Influence of temperature on effects of formulated moxidectin

We found temperature to moderate effects of formulated moxidectin on seed germination of all test species. Similarily, effects of a glyphosate-based herbicide formulation were found to be temperature-dependent in a study on the development of toad larvae [34]. In aquatic vertebrates, elevated water temperatures were found to increase the toxicity of the tested pesticide [34,67]. Temperature effects on chemical substances in living organisms are very complex, since temperature potentially acts on each chemical process and each plant tissue. Rising temperatures accelerate chemical processes [33], such as solubility or diffusion of a chemical substance. This might lead to quicker distribution of agricultural chemicals in the impacted organism and stronger cell damage. As explained above, stronger impacts of temperature on toxicity of formulated moxidectin can be expected especially if the seed coat is permeable to moxidectin and/or its adjuvants and the embryo tissues get into contact with the formulation ingredients already during seed imbibition. On the contrary, also molecule degradation processes might directly or indirectly be accelerated by increasing temperatures. And such an effect might mitigate or enhance toxic effects, depending on the toxicity of the degradation products. However, for our results we do not expect a direct effect of temperature on moxidectin degradation, since moxidectin is chemically stable within the temperature range studied [41].

We found that higher temperatures can boost effects of formulated moxidectin on MGT in some scenarios. For instance, in the 1:200 dilution of formulated moxidectin, germination speed was significantly lower when seeds are exposed to 30/20°C than to colder regimes in the species *C*. *jacea* and *G*. *mollugo*. In other scenarios, the warmest temperature regime did not lead to increased effects of formulated moxidectin, thus, hypothesis 2 was only partly confirmed by our results. Since we did not carry out measurements at the physiological level (e.g. enzymatic activies, amounts of heat shock proteins), we could not assess whether the 30/20°C regime caused heat stress to the studied seeds. Generally, an increase in temperature of >10°C above normal growing temperatures can be considered as heat stress [68]. However, whether heat stress is caused depends on several features, e.g., with respect to stressor: intensity and duration of temperature, and, with respect to organism: plant species and phenological stage [69]. For mesophilic plants, a heat-stress threshold of >35°C is specified [33]. Taking these information together, the studied species may have been stressed by the 30/20°C regime moderately at most.

The fact that the effects on germination found for a common agrigultural chemical depend on temperature is also of methodological significance. In most germination experiments, only one temperature regime is examined. Researchers should be aware that the choice of temperature regime can be decisive for the results and the assessment of the environmental toxicity of an agricultural chemical. In accordance to the literature [23], we found the highest germination percentages in the 20/10°C temperature regime, which is typical for the spring season in Central Europe, for all test species. If temperature is in the optimal range for the germination of a species, toxic effects of contaminants should be minimal, because temperature then does not act as an additional stressor to the seeds. Under field conditions, rised air temperatures are associated with a reduced water supply, i.e. higher evaporation rates and reduced soil water potentials. This combined abiotic effect might lead to higher stress levels than given in this study and to specific seed-contaminant responses and, thus, need further research.

## Concluding remarks and future perspectives

In this paper, we would like to raise awareness of the fact that temperature can have an influence on how toxic anthelmintic pharmaceuticals are for seed germination. More studies in this direction would be a worthwile issue for future research; specifically, we propose to take into account the following aspects:

■ Researchers studying toxicities of contaminants in germination experiments should carefully select the temperature regime to ensure comparability of results. We recommend choosing the optimal germination temperature for the test species, if temperature should not be studied as a factor imposing additional stress to the plant.
■ Larger sets of species should be tested to work out more precisely whether there are general patterns in the response of seeds to temperature influences on anthelmintic efficacy.
■ It should be elaborated whether it is primarily the active ingredient of a formulated drug which is temperature-dependent in its effects or whether it is an adjuvant or whether it is a combination of ingredients.

## Supporting information

S1 File The primary data of the germination experiment. (PDF)
