# Supplementary material for: Temperature moderates impact of formulated moxidectin on seed germination of three temperate grassland species
Source: PLoS One. 2022 Nov 21;17(11):e0277865. doi: 10.1371/journal.pone.0277865 (PMC9678283; doi:10.1371/journal.pone.0277865)
Supplement: S1 File — (PDF) [file pone.0277865.s001.pdf]

S1 File. The primary data of the germination experiment.

Temperature moderates impact of formulated moxidectin on seed germination of three temperate grassland species

Eichberg et al.

<u>Legend:</u>

Species: 1: Centaurea jacea, 2: Galium album, 3: Plantago lanceolata

Temperature regime (TR): 1: 15/5 °C, 2: 20/10 °C, 3: 30/20 °C

Solution: 1: control (purified water),

2: 1:800 dilution (d.) of Cydectin oral drench (0.1 % moxidectin), 3: 1:400 d., 4: 1:200 d., 5: 1:100 d.

No. of seedlings per counting date (3.5-day intervals)

Species	TR	Solution	Replicate	3.5	7	10.5	14	17.5	21	24.5	28	31.5	35
1	1	1	1	0	7	6	3	1	1	5	1	1	1
1	1	1	2	1	8	7	0	2	3	4	0	0	1
1	1	1	3	0	1	6	8	1	0	0	0	0	0
1	1	1	4	1	7	2	5	2	3	1	0	1	1
1	1	1	5	0	5	6	8	1	1	3	1	0	0
1	1	2	1	4	0	0	1	1	1	3	0	2	0
1	1	2	2	0	4	2	2	12	3	1	1	0	0
1	1	2	3	15	0	0	0	5	3	3	1	0	0
1	1	2	4	18	0	0	0	6	2	2	0	1	1
1	1	2	5	13	0	0	1	4	0	4	2	0	0
1	1	3	1	6	6	5	4	2	0	3	0	0	0
1	1	3	2	10	3	4	1	1	3	0	2	1	0
1	1	3	3	10	1	1	8	1	1	0	0	0	0
1	1	3	4	15	3	3	1	0	0	3	0	0	1
1	1	3	5	10	3	2	0	0	0	4	1	0	0
1	1	4	1	0	3	7	3	0	0	2	3	1	0
1	1	4	2	1	1	0	3	4	1	0	1	0	1
1	1	4	3	2	3	2	0	2	0	5	1	0	1
1	1	4	4	3	1	0	0	6	1	2	2	1	1
1	1	4	5	2	3	3	6	1	2	1	1	1	0
1	1	5	1	0	0	0	0	0	0	0	0	8	2
1	1	5	2	0	0	2	0	0	0	0	0	2	4
1	1	5	3	2	2	1	0	1	0	1	0	8	2
1	1	5	4	2	0	2	0	0	0	0	0	4	2
1	1	5	5	0	1	0	1	0	0	0	1	7	4
1	2	1	1	9	7	1	4	0	3	2	1	0	1
1	2	1	2	19	5	1	1	1	2	1	0	0	1
1	2	1	3	13	5	0	0	4	1	1	0	0	0
1	2	1	4	11	2	4	1	4	0	0	1	2	0
1	2	1	5	10	8	2	1	1	0	1	1	0	0
1	2	2	1	4	7	5	2	1	2	1	2	0	0
1	2	2	2	6	7	5	0	4	3	2	0	0	0
1	2	2	3	7	5	1	3	1	1	1	1	1	0
1	2	2	4	11	4	4	1	2	4	0	2	0	0
1	2	2	5	4	14	0	4	0	1	4	0	2	0
1	2	3	1	11	8	0	2	0	0	1	0	0	0
1	2	3	2	15	8	3	1	1	1	0	0	0	1
1	2	3	3	14	3	0	4	0	0	0	0	0	0

1	2	3	4	14	6	3	1	2	2	0	1	1	0
1	2	3	5	6	7	4	4	1	1	1	0	0	0
1	2	4	1	17	2	1	1	0	0	0	0	1	0
1	2	4	2	6	3	5	2	0	1	2	0	0	0
1	2	4	3	9	17	1	4	0	0	1	1	0	0
1	2	4	4	11	3	4	1	0	1	0	2	0	0
1	2	4	5	8	7	2	6	0	0	0	3	0	0
1	2	5	1	5	4	0	3	1	0	2	2	1	1
1	2	5	2	2	0	3	1	5	1	1	1	0	0
1	2	5	3	5	0	0	3	4	2	2	2	0	1
1	2	5	4	10	1	2	4	0	2	0	2	2	1
1	2	5	5	2	2	3	2	2	1	0	0	2	1
1	3	1	1	19	4	1	0	0	1	0	0	0	0
1	3	1	2	14	5	1	1	0	0	0	0	0	0
1	3	1	3	18	7	0	0	0	1	0	0	0	0
1	3	1	4	18	3	1	1	1	2	0	2	0	0
1	3	1	5	29	1	0	0	0	0	0	0	0	0
1	3	2	1	14	3	1	0	0	0	0	1	0	0
1	3	2	2	21	6	1	0	0	0	0	0	0	0
1	3	2	3	22	8	0	0	0	0	0	0	0	0
1	3	2	4	17	7	3	0	0	0	0	0	0	0
1	3	2	5	15	10	0	0	0	0	1	1	0	0
1	3	3	1	14	1	2	0	0	0	4	0	0	0
1	3	3	2	7	3	1	1	0	0	2	0	0	0
1	3	3	3	19	2	2	0	0	0	3	0	0	0
1	3	3	4	15	1	3	0	0	0	0	0	1	0
1	3	3	5	14	4	4	0	0	0	0	1	0	1
1	3	4	1	8	4	0	0	3	2	0	1	0	0
1	3	4	2	17	2	3	1	0	0	0	0	0	1
1	3	4	3	12	1	4	2	0	3	1	0	0	0
1	3	4	4	11	3	2	0	0	0	0	0	0	0
1	3	4	5	7	0	1	1	0	0	1	0	0	1
1	3	5	1	3	2	2	3	2	2	0	0	0	0
1	3	5	2	8	9	0	0	0	1	0	0	0	0
1	3	5	3	13	5	3	4	1	1	0	0	0	0
1	3	5	4	14	4	3	0	0	2	0	0	0	0
1	3	5	5	4	5	1	1	1	1	0	0	1	1
2	1	1	1	23	2	0	0	1	1	0	1	0	0
2	1	1	2	23	3	1	0	1	0	1	1	0	0
2	1	1	3	19	3	3	0	2	2	0	1	0	0
2	1	1	4	18	4	3	1	1	2	1	1	0	1
2	1	1	5	18	4	2	0	2	0	0	1	0	0
2	1	2	1	22	4	1	1	1	0	1	0	0	0
2	1	2	2	23	0	1	2	0	0	0	0	0	0
2	1	2	3	26	1	2	1	1	1	0	0	0	0
2	1	2	4	17	6	2	0	0	1	1	0	0	0
2	1	2	5	27	1	3	1	2	0	0	0	0	0
2	1	3	1	19	3	2	0	1	1	1	0	0	1
2	1	3	2	24	3	3	0	0	0	0	0	1	0
2	1	3	3	16	1	4	1	2	0	1	0	0	0

2	1	3	4	18	3	2	3	1	0	1	0	1	0
2	1	3	5	20	2	4	0	3	1	0	0	0	0
2	1	4	1	14	2	1	0	1	0	1	0	1	0
2	1	4	2	23	1	1	0	0	1	0	1	0	0
2	1	4	3	24	0	1	0	1	0	3	0	0	0
2	1	4	4	18	0	1	1	1	1	0	0	0	0
2	1	4	5	21	0	2	0	0	0	0	1	1	0
2	1	5	1	24	1	1	0	0	0	0	0	0	0
2	1	5	2	21	0	0	0	0	0	1	1	0	0
2	1	5	3	23	0	0	1	0	0	0	0	0	0
2	1	5	4	21	0	0	0	1	0	0	0	0	0
2	1	5	5	20	1	0	0	0	0	0	0	0	1
2	2	1	1	29	9	3	0	1	1	0	0	0	0
2	2	1	2	22	12	2	1	0	0	0	0	0	1
2	2	1	3	30	8	3	0	0	1	0	3	1	0
2	2	1	4	31	9	1	0	2	0	0	0	0	0
2	2	1	5	32	12	2	1	0	0	0	0	0	0
2	2	2	1	33	10	0	1	0	0	0	0	0	1
2	2	2	2	21	11	1	2	0	1	0	0	0	1
2	2	2	3	28	5	5	1	1	1	0	1	1	0
2	2	2	4	29	10	4	0	0	0	0	0	0	0
2	2	2	5	25	9	3	1	1	0	0	0	1	1
2	2	3	1	15	3	2	1	6	7	0	1	0	0
2	2	3	2	27	2	0	3	4	0	1	1	0	0
2	2	3	3	25	4	3	2	0	2	1	0	0	0
2	2	3	4	17	3	7	1	0	1	0	0	1	0
2	2	3	5	24	5	2	2	0	1	1	0	1	0
2	2	4	1	22	1	0	1	0	0	0	0	0	0
2	2	4	2	16	5	1	0	1	6	4	1	0	0
2	2	4	3	21	1	1	2	1	0	1	0	0	0
2	2	4	4	18	2	0	4	0	3	3	1	0	0
2	2	4	5	22	2	0	0	1	1	1	0	0	0
2	2	5	1	23	0	0	0	0	0	1	2	1	0
2	2	5	2	18	2	0	0	0	2	3	0	0	0
2	2	5	3	20	1	2	2	0	0	1	1	0	1
2	2	5	4	25	3	0	0	0	0	1	1	0	1
2	2	5	5	23	1	0	1	0	0	0	3	1	0
2	3	1	1	34	5	1	1	1	0	0	1	0	0
2	3	1	2	37	4	1	1	0	0	0	1	0	1
2	3	1	3	33	2	0	0	0	0	1	0	1	0
2	3	1	4	33	4	1	0	0	1	1	0	0	1
2	3	1	5	32	6	1	3	0	2	0	0	0	0
2	3	2	1	27	7	1	3	0	2	1	0	1	1
2	3	2	2	33	4	2	1	1	0	0	0	0	1
2	3	2	3	26	8	4	2	0	0	1	0	0	0
2	3	2	4	25	9	3	2	1	2	2	0	0	0
2	3	2	5	28	7	0	4	0	1	1	0	0	1
2	3	3	1	26	0	1	0	0	4	2	2	0	1
2	3	3	2	20	0	0	2	0	3	0	2	1	2
2	3	3	3	20	2	3	1	3	3	3	1	0	0

2	3	3	4	19	7	1	1	0	1	1	2	1	3
2	3	3	5	27	5	2	2	0	0	4	0	1	1
2	3	4	1	18	1	0	0	0	0	3	1	1	0
2	3	4	2	23	1	0	0	0	1	2	3	0	0
2	3	4	3	14	0	0	0	0	0	0	6	2	0
2	3	4	4	14	0	0	0	0	3	4	2	0	0
2	3	4	5	24	0	0	0	0	3	1	2	0	2
2	3	5	1	14	1	0	0	0	1	0	0	1	0
2	3	5	2	19	0	0	0	0	0	1	0	0	0
2	3	5	3	17	2	0	0	0	0	3	2	0	1
2	3	5	4	20	0	0	0	0	0	1	1	0	0
2	3	5	5	19	2	0	0	1	0	1	0	0	0
3	1	1	1	0	1	8	9	5	3	0	2	0	0
3	1	1	2	0	3	18	4	5	2	2	0	0	0
3	1	1	3	0	5	16	9	0	0	3	0	2	0
3	1	1	4	0	3	17	0	7	3	2	0	0	0
3	1	1	5	0	10	9	3	0	5	2	1	0	0
3	1	2	1	0	0	5	5	10	8	0	1	4	2
3	1	2	2	1	10	0	4	3	4	1	1	0	0
3	1	2	3	3	7	8	0	3	2	1	0	1	2
3	1	2	4	0	0	10	4	5	2	3	6	2	4
3	1	2	5	0	5	4	2	2	1	2	0	2	3
3	1	3	1	0	5	4	0	0	1	1	2	0	0
3	1	3	2	3	3	0	0	1	0	1	2	1	0
3	1	3	3	0	0	0	0	1	0	1	0	0	2
3	1	3	4	0	1	0	1	0	0	0	2	0	1
3	1	3	5	0	0	0	4	0	0	1	2	0	0
3	1	4	1	0	0	0	0	2	4	2	2	2	1
3	1	4	2	0	0	0	1	2	2	7	5	4	4
3	1	4	3	0	0	0	0	4	2	1	3	1	0
3	1	4	4	0	0	1	1	1	0	0	0	0	1
3	1	4	5	0	2	1	1	3	1	4	0	2	0
3	1	5	1	0	0	0	2	0	0	2	8	10	3
3	1	5	2	0	0	0	1	0	0	3	5	4	8
3	1	5	3	0	0	0	0	0	1	5	6	15	1
3	1	5	4	0	0	0	1	0	0	3	0	4	8
3	1	5	5	0	0	0	0	0	2	3	3	15	12
3	2	1	1	6	1	0	1	0	0	0	9	3	6
3	2	1	2	3	4	3	0	1	0	6	12	2	2
3	2	1	3	7	4	0	1	0	0	4	2	0	0
3	2	1	4	4	2	1	2	1	1	0	0	2	2
3	2	1	5	1	2	3	3	0	0	0	7	9	4
3	2	2	1	3	7	1	7	2	1	0	5	3	4
3	2	2	2	4	8	4	5	2	1	3	6	5	2
3	2	2	3	1	9	3	0	2	0	0	0	1	0
3	2	2	4	2	2	0	5	4	2	2	1	0	0
3	2	2	5	1	4	2	7	3	0	2	0	0	4
3	2	3	1	4	9	8	12	2	1	0	0	0	0
3	2	3	2	1	12	9	4	3	0	0	0	0	0
3	2	3	3	2	8	15	9	7	1	0	0	0	0

3	2	3	4	1	10	8	5	4	1	0	0	0	0
3	2	3	5	3	7	9	9	1	2	0	0	0	0
3	2	4	1	1	7	3	0	3	0	0	0	0	0
3	2	4	2	0	2	3	8	10	3	0	0	0	0
3	2	4	3	0	1	2	6	0	5	0	0	0	0
3	2	4	4	0	1	0	10	3	2	1	2	0	0
3	2	4	5	0	0	6	10	6	2	0	1	1	0
3	2	5	1	0	0	0	0	0	6	9	9	6	1
3	2	5	2	0	0	0	0	0	0	3	0	7	4
3	2	5	3	0	0	0	0	1	1	6	7	11	7
3	2	5	4	0	0	0	0	1	0	4	6	4	2
3	2	5	5	0	0	0	0	1	2	4	6	7	6
3	3	1	1	14	3	0	0	0	0	1	0	2	0
3	3	1	2	11	4	0	0	0	0	3	3	0	0
3	3	1	3	11	7	0	0	0	0	2	1	1	1
3	3	1	4	13	8	0	1	0	0	1	0	0	1
3	3	1	5	16	9	0	0	0	0	0	1	1	0
3	3	2	1	3	10	2	5	2	0	0	0	0	1
3	3	2	2	5	10	3	2	0	0	0	0	0	0
3	3	2	3	3	10	6	1	0	0	1	1	0	1
3	3	2	4	3	3	1	1	0	1	0	0	0	0
3	3	2	5	4	6	3	1	1	1	1	0	0	0
3	3	3	1	2	8	7	1	0	6	2	0	0	1
3	3	3	2	1	0	2	4	3	2	1	0	0	0
3	3	3	3	0	0	7	8	1	0	1	0	1	0
3	3	3	4	0	1	3	7	2	2	2	3	0	0
3	3	3	5	1	5	10	2	2	1	1	2	0	1
3	3	4	1	0	10	5	4	3	0	0	0	0	0
3	3	4	2	0	0	2	11	3	2	1	2	0	1
3	3	4	3	0	0	0	14	10	6	0	0	0	0
3	3	4	4	0	1	5	10	3	3	1	0	0	2
3	3	4	5	0	10	8	2	0	3	3	0	0	0
3	3	5	1	0	0	0	2	24	7	2	0	2	0
3	3	5	2	0	0	0	2	12	7	3	2	2	2
3	3	5	3	0	0	0	2	9	18	3	1	0	0
3	3	5	4	0	0	0	3	18	6	1	0	0	0
3	3	5	5	0	0	0	1	19	6	2	0	0	1
